# Pathophysiological clinical features of an infant with hypertension secondary to multicystic dysplastic kidney: a case report

**DOI:** 10.1186/s12882-021-02249-6

**Published:** 2021-02-05

**Authors:** Keisuke Sugimoto, Takuji Enya, Kensuke Joh, Kohei Miyazaki, Tomoki Miyazawa, Rina Ohshima, Satoshi Marutani, Takemura Tsukasa, Mitsuru Okada

**Affiliations:** 1 Department of Pediatrics, Kindai University Faculty of Medicine, 377-2 Ohno-higashi, Osakasayama-shi, Osaka, 589-8511 Japan; 2 Department of Pathology, Jikei University School of Medicine, Minato-ku, Tokyo, Japan; 3 Department of Pediatrics, Kushimoto Municipality Hospital, Higashimuro-gun, Wakayama, Japan

**Keywords:** Multiple cystic dysplastic kidney, Nephrectomy, Hypertension

## Abstract

**Background:**

The association of hypertension with congenital renal hypoplasia has been established. We report a case of an infant who underwent nephrectomy for hypertension.

**Case presentation:**

Magnetic resonance imaging for the mother revealed fetal renal masses, and fetal multicystic dysplastic kidney was suspected. Following birth, the baby developed hypertension. Numerous investigations revealed that the left kidney was non-functional, and she was initiated on benazepril hydrochloride. However, because the drug response was poor, the left kidney was removed at the age of 7 months. Examination of the renal specimen revealed abrupt transition from normal to atrophic cortex with lobar atrophy and cysts. Tubular atrophy, marked abnormal blood vessels with wall thickening, gathered immature glomeruli, and parenchymal destruction were observed. Renin was partially localized in the proximal tubules and the parietal epithelium of the Bowman’s capsule in the immature glomeruli. We speculated that an abnormal vascular structure and irregular renin localizations may be the cause of hypertension. Serum renin and aldosterone levels gradually reduced post-surgery, reaching normal levels on the 90th postoperative day. A long follow-up is needed due to the possibility of the child developing hypertension in the future.

**Conclusion:**

This is a case of an infant with MCDK, which discusses the clinicopathological features based on the pathophysiological analysis, including renin evaluation.

## Background

The prevalence of hypertension in children and adolescents is 1–5% [1, 2]. Unlike adult hypertension, which is usually primary, childhood hypertension is generally secondary to an underlying disorder [3, 4]. Renal diseases are the main causes of severe hypertension in children and adolescents [4]. A previous study reported that perinatal risk factors and secondary causes, such as renal disease, are responsible for neonatal hypertension [5]. Severe hypertension may result from different causes such as cardiac output dysfunction and peripheral vascular resistance [4].

Multicystic dysplastic kidney (MCDK) is one of the common renal cystic diseases that are identified as congenital anomalies of the kidney and urinary tract. It is estimated to occur in 1 in every 4300 live births [6, 7]. While the pathogenesis of this disorder remains unclear, it is considered to involve failure of the ureteric bud to integrate and branch appropriately into the metanephros during development in early childhood [8]. Because MCDK tends to involute (in appropriately 40% of cases), the prognosis of simple MCDK (defined as unilateral renal dysplasia without additional genitourinary abnormalities) is generally good [9]. However, several studies have reported a risk of hypertension in patients with MCDK [10, 11]; for instance, the Multicystic Kidney Registry reported mild hypertension in 4 out of 260 individuals (1.5%) with MCDK [12]. It is believed that distortion of the renal architecture leads to structural damage and tubular dysfunction, and results in activation of the renin-angiotensin-aldosterone system (RAAS) in polycystic kidney disease. Herein, we report a case of an infant with MCDK, and describe its clinicopathological features based on the pathophysiological analysis (including renin evaluation).

## Case presentation

**Fig. 1:**
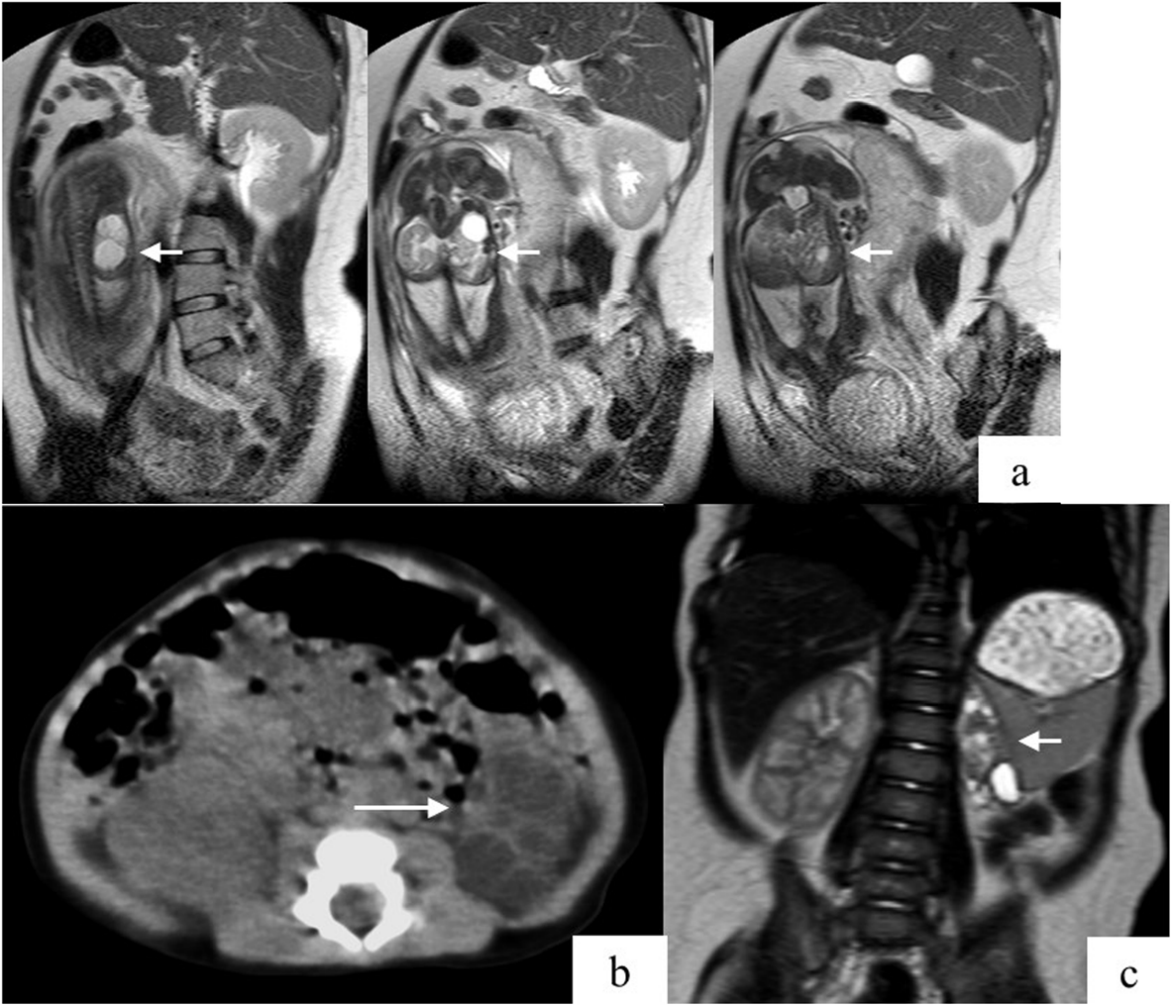
Magnetic resonance imaging. **a** Prenatal magnetic resonance imaging shows fetal cystic lesions of the left kidney (short arrow). **b** Multiple cystic lesions on the left kidney are noted on computed tomography (long arrow). **c** Magnetic resonance imaging shows atrophic left kidney with multiple cysts (short arrow)

**Fig. 2:**
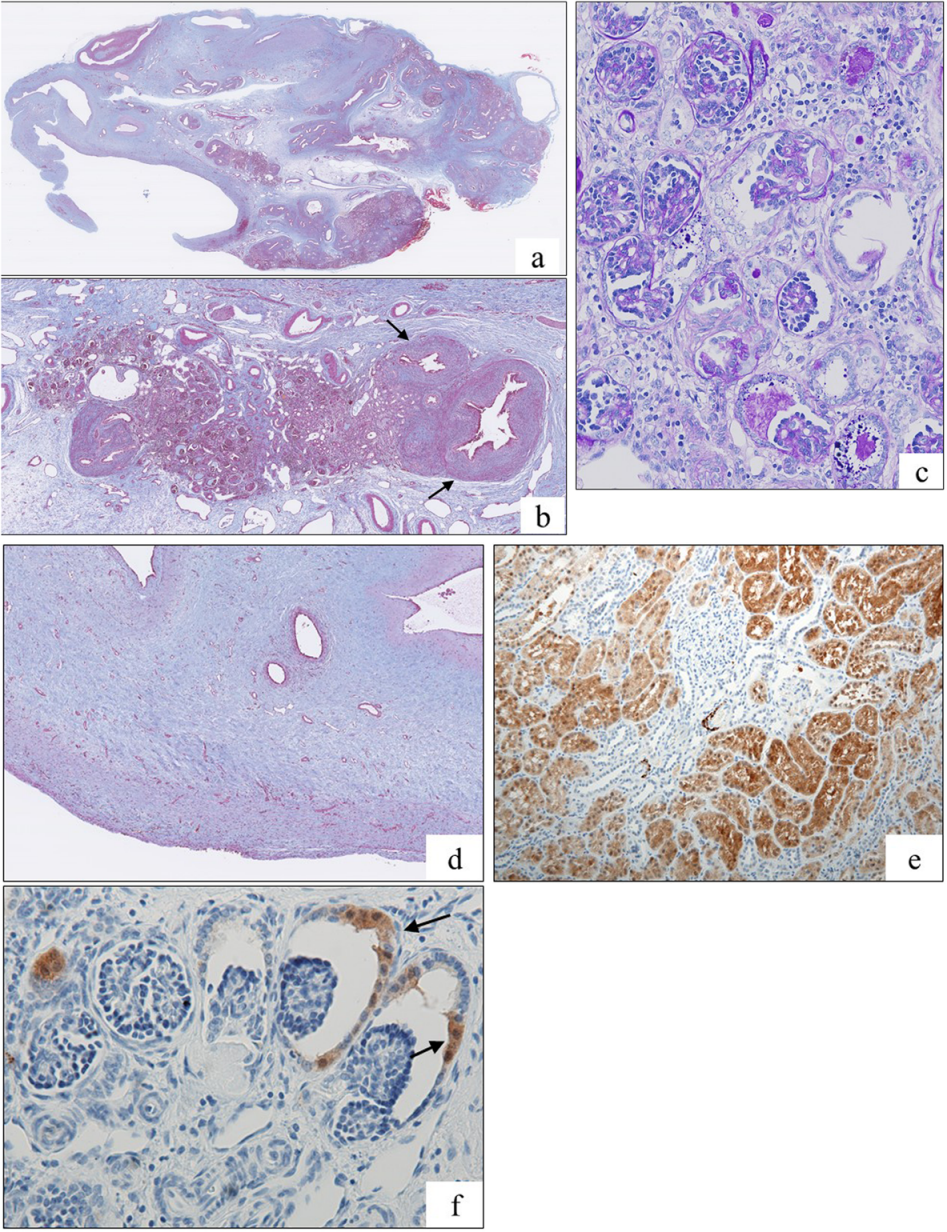
**a** Light microscopy of the entire cross-section reveals abrupt transition from normal to atrophic cortex with lobar atrophy (Masson-trichrome staining, 4×). **b** Tubular atrophy, marked abnormal blood vessels with wall thickening (short arrow), gathered glomeruli, and parenchymal destruction are observed (Masson-trichrome staining, 20×). **c** Diffuse immature glomerulus with interstitial fibrosis is observed (Periodic acid-Schiff staining, 250×). **d** Fibromuscular tissue proliferation is observed around the renal calyx (Masson-trichrome staining, 40×). **e**, **f** Renin localized partially in the proximal tubules and the parietal epithelium of the Bowman’s capsule in the immature glomeruli (short arrow) (Renin antibody staining, 200×)

The patient was a 13-year-old Japanese girl with no family history of renal disease or hypertension. There was no history of medication with non-steroidal anti-inflammatory drugs or RAAS inhibitors during her mother’s pregnancy. Prenatal ultrasonography revealed an abdominal mass lesion in the fetus. At around 26 weeks of gestation, the mother underwent magnetic resonance imaging (MRI), which revealed fetal renal mass lesions indicative of MCDK (Fig. 1a). The patient was naturally delivered at full term; her birth weight was 3076 g. General laboratory examinations did not reveal any clinical abnormalities; therefore, the mother and patient were discharged. However, the patient presented with increasing blood pressure around 1 month after birth, and gradually developed hypertension. On admission at 6 months of age, her development was observed to be normal. Physical examination also revealed normal weight and height without surface malformation. Blood pressure was checked multiple times and was 110/80 mmHg on the first day of admission. Urinalysis demonstrated no proteinuria, hematuria, and glycosuria. Serum sodium (138 mEq/L), potassium (4.8 mEq/L) and calcium (11.7 mg/dL) results and the serum creatinine level (0.25 mg/dL) were normal. Elevated plasma renin activity (17.1 ng/mL/hr.; normal: 0.2–2.3 ng/mL/hr) and serum aldosterone levels (731 pg/mL; normal: 29.2–159.0 pg/mL) were observed. Brain MRI demonstrated no signs of microvascular lesions. Abdominal ultrasonography revealed left renal hypoplasia without scarring on the kidney surface. MRI revealed an atrophic left kidney with multiple cysts (Fig. 1c) and no sign of adrenal enlargement. Renal DTPA scintigraphy with Tc-99 m dimercaptosuccinic acid revealed differential glomerular filtration rates (GFR) of 68 mL/min and 7 mL/min for the right and left kidney, respectively, suggesting that the left kidney was nonfunctional. These findings suggested small vessel renovascular hypertension, although neither large vessel renovascular stenosis nor obstruction was observed during MRI. She was initiated on benazepril hydrochloride (0.03 mg/kg/day); however, because the response to the drug was poor, the left kidney was removed at the age of 7 months. Analysis of the renal specimen revealed interstitial fibrosis, cell invasion, and marked arterial wall thickening; glomeruli and tubular formation were absent in the same region (Fig. 2a and b). Diffuse immature glomerulus was observed in the cortex area that was retained partially (Fig. 2c). Fibromuscular tissue proliferation was observed around the renal calyx (Fig. 2d). Immunohistochemical staining with anti-renin antibody revealed that renin was partially localized in the proximal tubules and the parietal epithelium of the Bowman’s capsule in the immature glomeruli (Fig. 2e and f). The blood pressure normalized after the nephrectomy. The renin and aldosterone levels also gradually reduced to 3.4 ng/mL/hr. and 94.2 pg/mL, respectively, reaching normal levels on the 90th postoperative day. The serum creatinine level and eGFR at the latest examination were 0.56 mg/dL and 129 ml/min/1.73 m2, respectively. The baby undergoes routine check-up with blood pressure monitoring and is currently not on any medications.

### Pathologic analysis

Briefly, the kidney specimens were fixed in 10% neutral buffer formalin, and were embedded in paraffin for light microscopy analysis with routine staining with hematoxylin and eosin, periodic acid-Schiff, Masson’s trichrome, and periodic acid-methenamine-silver stains. For renin staining, Discovery Ultra Autostainer (Ventana Medical Systems, Inc. Tucson, AZ, USA) with I-VIEW DAB kit was used on paraffin-embedded specimens. Following deparaffinization and antigen retrieval (CC1 Mild), mouse anti-renin antibodies (clone 411,507) (R & D Systems) were used as the primary antibody with × 100 dilution for 32 min. For a negative control, normal mouse IgG1 (R & D Systems) was used on the same specimen. In order to compare the present case with other cases concerning the distribution of renin at the vascular pole of the glomerulus, a case of diabetes mellitus glomerulopathy at the same age without hypertension was used.

## Discussion and conclusions

MCDK is the most common cause of cystic disease in children and is one of the most commonly detected anomalies on prenatal ultrasound [13]. It is characterized by replacement of the normal kidney tissue with numerous cysts, undifferentiated epithelium, and primitive ducts surrounded by fibromuscular connective tissue [14, 15]. In the present case, renal MRI detected renal cyst formation, leading us to diagnose the infant with MCDK, which is a potential cause of hypertension after birth. A 2005 meta-analysis revealed that the risk of hypertension among such children was actually less than that observed in the general population (0.54% vs. 1–4.5% between the ages of four and adolescence) [6]. Eickmeyer et al. reported that 9 of the 301 patients analyzed developed hypertension throughout the follow-up period, constituting the 3% incidence of hypertension observed [11]. Furthermore, the occurrence of hypertension in the present MCDK population was found to be consistent with that quoted in the general pediatric population, and is unlikely to warrant aggressive subspecialty follow-up for uncomplicated MCDK. Multiple studies on children with MCDK have revealed similar results [16–18].

One of the mechanism of hypertension in some cases include elevated renin levels, while in some cases, renin levels are normal [19–21]. In our case, both renin and aldosterone levels were remarkably elevated after birth. Nephrectomy performed for hypertension is not always curative, and there are no specific guidelines for performing a nephrectomy for MCDK. In a retrospective study from Turkey, 20 cases with MCDK underwent nephrectomy; the indication for nephrectomy was hypertension in four patients, abdominal mass in six patients, and recurrent urinary infection in the remaining 10 patients [22]. Conversely, Oliveria et al. reported the case of a newborn girl with MCDK whose blood pressure levels improved spontaneously at 10 months of age [23]. In our case, nephrectomy was performed because the response to an antihypertensive drug was poor. The postoperative clinical course was good in our case as well as in other cases that underwent nephrectomy. Several studies have reported the resolution of hypertension following the removal of a unilateral poorly or non-functioning kidney (including MCDK), permitting cessation or diminution of antihypertensives [24–26]. In our case, the blood pressure normalized with decreasing renin and aldosterone levels after nephrectomy, as in previous reports. Urinary malformations in MCDK patients consist of vesicoureteral reflux (VUR), ureteropelvic junction obstruction, and kidney stones. Contralateral VUR is the most common urological anomaly in MCDK, and ipsilateral VUR might also be present [27]. In our case, existence of VUR was unclear because voiding cystourethrography was not performed. MRI revealed that the renal abnormality existed during pregnancy, suggesting that VUR may not be the main cause of segmental hypoplasia. Even in cases without VUR, unilateral renal agenesis including MCDK is suggestive of immature kidney development, such as a reduced number of nephrons [28, 29]. Furthermore, hypertension is caused by anomalies of the kidney; remarkable arterial wall thickening in particular leads to an increase in the peripheral vascular resistance [19]. In our case, a diffuse immature glomerulus was observed in the cortex area containing interstitial fibrosis and multicysts, which is consistent with the findings of previous reports. Small arteries were observed to be connected to the interlobular arteries, extending like vines in the fibrous areas. Furthermore, most of the renal parenchyma was replaced by collagen fibers. The site of renin production is usually the vascular polar macula densa; in our case, renin was partially localized in the proximal tubules and the parietal epithelium of the Bowman’s capsule in the immature glomeruli. Abdulhannan et al. reported a similar case of a 2-month-old girl; however the serum renin and the renin expression in the renal specimen were not evaluated [30]. Furthermore, proximal tubular production of renin in the transgenic mice can result in a systemic increase in arterial pressure [31]. We demonstrated that an abnormal renal structure (including abnormal small arteries) and irregularly renin localizations in the proximal tubules and the parietal epithelium of the Bowman’s capsule in the immature glomeruli. These data may help to explain that these abnormalities account for the child’s hypertension.

In conclusion, the risk of chronic renal insufficiency or end-stage renal disease at 5 years of age in simple MCDK patients is less than that of complex MCDK [9]. The infant in our case did not present with urinary tract infection, proteinuria, or renal insufficiency. However, although the blood pressure is currently stable, a long follow-up is needed due to the possibility of developing hypertension in the future, because a contralateral renal tissue is not always normal [32, 33].

## Data Availability

Not applicable.

## Abbreviations

GFR: Glomerular filtration rate
MCDK: Multiple cystic dysplasia kidney
MRI: Magnetic resonance imaging
VUR: Vesicoureteral reflux
